# Multi-Drug Resistance Mediated by Class 1 Integrons in *Aeromonas* Isolated from Farmed Freshwater Animals

**DOI:** 10.3389/fmicb.2016.00935

**Published:** 2016-06-15

**Authors:** Yuting Deng, Yali Wu, Lan Jiang, Aiping Tan, Ruiquan Zhang, Li Luo

**Affiliations:** ^1^Key Laboratory of Fishery Drug Development, Ministry of Agriculture, Key Laboratory of Aquatic Animal Immune Technology, Pearl River Fisheries Research Institute, Chinese Academy of Fishery SciencesGuangzhou, China; ^2^Office of Aquaculture Technology Extension, Foshan Institute of Agricultural SciencesFoshan, China

**Keywords:** *Aeromonas*, multidrug resistance, class 1 integron, resistance genes, freshwater animal

## Abstract

*Aeromonas* is regarded as an important pathogen of freshwater animals but little is known about the genetics of its antimicrobial resistance in Chinese aquaculture. The aim of this study was to investigate the presence of integrons and characterize multidrug resistant *Aeromonas* spp. isolated from diseased farmed freshwater animals. These animal samples included fish, ornamental fish, shrimp, turtles, and amphibians which were collected from 64 farms in Guangdong province of South China. One hundred and twelve *Aeromonas* spp. isolates were examined for antimicrobial resistance phenotypes and the presence of class 1 integron sequences. Twenty-two (19.6%) of these isolates carried a class 1 integron comprising six different gene insertion cassettes including *drfA12-orfF-aadA2, drfA12-orfF, aac(6*′*)-II-bla*_OXA-21_*-cat3, catB3, arr-3*, and *dfrA17*. Among these, *drfA12*-*orfF*-*aadA2* was the dominant gene cassette array (63.6%, 14/22) and this is the first report of *aac(6*′*)-II-bla*_OXA-21_*-cat3* in an *Aeromonas hydrophila* isolate from a Chinese giant salamander (*Andrias davidianus*). All the integron-positive strains were resistant to more than five agents and 22 contained other resistance genes including *bla*_CTX-*M*-3_, *bla*_TEM-1_, *aac(6*′*)-Ib-cr*, and *tetA*. All integron-positive isolates also contained mutations in the quinolone resistance determining regions (QRDR). Our investigation demonstrates that freshwater animals can serve as a reservoir for pathogenic *Aeromonas* strains containing multiple drug-resistance integrons. This data suggests that surveillance for antimicrobial resistance of animal origin and a prudent and responsible use of antimicrobials in aquaculture is necessary in these farms.

## Introduction

The genus *Aeromonas* is regarded as an important pathogen of freshwater animals. Motile aeromonads including *A. hydrophila, A. veronii, A. caviae*, and *A. sobria* are considered facultative pathogens and can infect fish as well as shrimp, reptiles, amphibians, and other aquatic species (Janda and Abbott, 2010). *Aeromonas* infections have been linked to major die-offs and fish kills around the globe over the past decade resulting in enormous economic losses. Furthermore, *Aeromonas* can cause disease in warm-blooded animals and can even infect humans *via* consumption of contaminated food or water (Janda and Abbott, 2010).

Treatment and prevention of diseases in animal husbandry including livestock and aquaculture have been relying on the extensive use of antimicrobials. This practice has been undoubtedly increased the frequency of antibiotic-resistant bacterial strains and this has been widely documented. Antimicrobial resistance in bacterial populations can result from clonal selection under antimicrobial selective pressure or through horizontal gene transfer. Mobile genetic elements such as plasmids, integrons, and transposons contribute to a wider dissemination of genetic resistance determinants among bacteria (Boerlin and Reid-Smith, 2008). Of these, the mobile integron encoded integrases can recombine gene cassettes and are primarily involved in the spread of antimicrobial resistance genes which contributed to multidrug-resistance (Mazel, 2006). The class 1 integron is the most common integron type and has been found in a wide range of gram-negative bacteria from various sources (Hall, 2012). Previous studies have found integron in *Aeromonas* spp. mainly belong to the class 1 and carried various antimicrobial resistance gene cassettes. The most often found resistance gene cassettes contained several aminoglycoside resistance gens *aadA1, aadA2*, and the trimethoprim resistance gene *dfrA1* (Piotrowska and Popowska, 2015).

Motile *Aeromonas* species are important aquatic pathogens and are considered a public health concern. Several types of fluoroquinolones and sulfonamides have been approved for treatment of these infections and are frequently used in veterinary medicine [Ministry of Agriculture of the People's Republic of China (MOA), 2010; Su et al., 2011]. Consequently, resistance to antimicrobial has been widely spread in aquaculture environments. Integrons-inserted gene cassettes have been reported in widespread *Aeromona*s species isolated from diseased fish (Piotrowska and Popowska, 2014). However, only a few studies, to date, addressed the prevalence of class 1 integron in Chinese aquaculture (Feng et al., 2011; Su et al., 2011; Jiang et al., 2012).

In this study, we investigated the distribution of integrons among aeromonads isolated from farmed diseased freshwater animals. We documented the presence of resistance genes and whether mutations of quinolone resistance determining regions (QRDR) were present among the integron-positive isolates. We evaluated the potential of multi-drug resistant aeromonads in these animals as a public health risk.

## Materials and methods

### Bacterial isolation and genetic identification

A total of 112 *Aeromonas* species isolated from diseased freshwater animal samples, including 67 fish from 37 farms, 7 ornamental fish from 6 farms, 12 shrimp from 6 farms, 21 turtles from 12 farms, and 5 amphibians from 3 farms in Guangdong province. All samples were collected at fisheries hospitals of the Pearl River Fisheries Research Institute from November 1995 to February 2012. All these freshwater animals were raised as food animals, pets or for other purposes. Gills, body surfaces, and livers were aseptically swabbed using sterile cotton buds and inoculated into Luria–Bertani broth for pre-enrichment at 30 ± 2°C for 18–24 h. The enriched cultures were streaked on Rimler–Shotts agar and incubated at 30 ± 2°C for 18–24 h. Yellow, oxidase-positive colonies were isolated and were presumptively considered as *Aeromonas* species. One colony per sample with typical *Aeromonas* morphology was selected and identified by API 20E biochemical tests (BioMérieux, France). For further identification, polymerase chain reaction (PCR) amplification of 16S rRNA gene and *gyrB* gene was performed as described in previous studies (Borrell et al., 1997; Yáñez et al., 2003). Taxonomic identification of the DNA sequences was performed using BLAST in GenBank (http://blast.ncbi.nlm.nih.gov/).

### Antimicrobial susceptibility testing

All strains were evaluated for resistance to 14 antimicrobials by the disk diffusion method using commercially available disks (Oxoid, U.K.). These drugs included ampicillin (10 μg), cefotaxime (30 μg), sulfonamides (300 μg), trimethoprim/sulfamethoxazole (1.25/23.75 μg), rifampin (5 μg), nalidixic acid (30 μg), ciprofloxacin (5 μg), norfloxacin (10 μg), ofloxacin (5 μg), tetracycline (30 μg), doxycycline (30 μg), streptomycin (10 μg), amikacin (30 μg), and chloramphenicol (30 μg) discs. *Escherichia coli* ATCC 25922 was used for quality control. The results were evaluated as susceptible (S), intermediate (I), and resistant (R) based on the interpretative criteria from the [Clinical and Laboratory Standard Institute (CLSI), 2006a,b].

### DNA extraction and PCR amplification of integrase genes

DNA prepared by the whole cell boiled lysate protocol was used for PCR templates. All isolates were screened for the presence of *intI1, intI2, intI3*. All the *intI1*-positive strains were also amplified for *sul1* and *qacE*Δ*1* fragments by PCR using primers described previously (Sandvang et al., 1997; Reyes et al., 2003). The PCR primers used in this study are presented in Supplementary Table 1.

### Amplification and sequencing of gene cassette regions

Gene cassette arrays of *intI1*-positive strains were determined by PCR using primers described previously (Lévesque et al., 1995; Supplementary Table 1). PCR products were directly sequenced and the DNA sequences were analyzed using BLAST (http://blast.ncbi.nlm.nih.gov/).

### PCR amplification of resistance genes and quinolone resistance determining regions (QRDR)

To characterize other resistance genes of the integron-positive isolates, extended spectrum beta-lactamase (ESBL) genes (*bla*_TEM_, *bla*_CTX_), tetracycline resistance genes (*tetA, tetC*, and *tetE*), and plasmid-mediated quinolone resistance (PMQR) genes [*qnrA, qnrB, qnrS, aac(6*′*)-Ib-cr*, and *qepA*] were also screened by PCR amplification as previously described (Liu et al., 2008). The sequencing primers are presented in Supplementary Table 1. Sequences were aligned with known sequences in the NCBI database using BLAST and are reported below as Genbank reference numbers.

The mutation within the QRDRs of *gyrA* and *parC* in integron-positive isolates were also determined as previously described (Giraud et al., 2004).

## Results

### Identification of *Aeromonas* spp. from different aquatic animals

Base on the biochemical and genetic characteristics, 112 *Aeromonas* isolates were identified up to the species level. They belonged to eight different species of *Aeromonas* (*A. hydrophila, A. veronii, A. caviae, A. sobria, A. dhakensis, A. jandaei, A. trota*, and *A. media*; Table 1). Over all, *A. hydrophila* was the most prevalent species, and were most frequently isolated from all kinds of animals analyzed, indicating that it is the predominant species in aquatic pathogens.

**Table 1:** **Prevalence of ***Aeromonas*** spp. in different aquatic animals**.

***Aeromonas* spp**.	**Sources**				
	**Fish**	**Ornamental fish**	**Shrimp**	**Turtles**	**Amphibians**
*A. hydrophila* (*n* = 62)	34	5	4	16	3
*A. veronii* (*n* = 23)	17	2		3	1
*A. caviae* (*n* = 7)	4		2	1	
*A. sobria* (*n* = 6)	5		1		
*A. dhakensis* (*n* = 4)	2		1	1	
*A. trota* (*n* = 4)	1		3		
*A. jandaei* (*n* = 3)	3				
*A. media* (*n* = 1)					1
Unidentified (*n* = 2)	1		1		
Total (*n* = 112)	67	7	12	21	5

### Detection and characterization of integron and gene cassettes

Among 112 *Aeromonas* strains isolated from different farmed freshwater animals, 22 (19.6%) isolates were positive for *intI1* while *intI2* and *intI3* were not detected in any of the isolates. All of integron-positive isolates also contained *sul1* and *qacE*Δ*1* which indicated that these isolates contained structurally complete integrons. Considering the different sources, *A. hydrophila* (81.8%, 18/22) was the most frequently isolated species carrying integrons, which were more prevalent in *Aeromonas* spp. isolated from fish and turtles (Table 2).

**Table 2:** **Characteristic of 22 integron-positive ***Aeromonas*** isolates**.

**Strain**	***Aeromonas* spp**.	**Year**	**Species**	**Farm**	**Class 1 integron gene cassettes**	**Resistance genes**	**QRDR mutation**		**Antimicrobial resistance profile**
							**GyrA**	**ParC**	
A15	*A. hydrophila*	1998	Fish *(Channa argus)*	FF6	*dfrA17*	*tetA*	Ile83	Ser80	AMP\S3\SXT\RD\NA\TE\C\S
A16	*A. hydrophila*	1998	Fish *(C. argus)*	FF6	*dfrA17*	*tetA*	Ile83	Ser80	AMP\S3\SXT\RD\NA\TE\C\S
A36	*A. hydrophila*	2004	Ornamental fish *(Crypinus carpiod)*	OF1	*drfA12-orfF-aadA2*		Ile83	Ile80	AMP\S3\SXT\RD\NA\OFL\TE\DO\C\S
A25	*A. hydrophila*	2004	Turtle *(Chelydra serpentine)*	TF2	*drfA12-orfF-aadA2*	*bla*_CTX−M−3_, *bla*_TEM−1_, *aac(6′)-Ib-cr*	Ile83	Ile80	AMP\CTX\S3\SXT\RD\NA\CIP\NOR\OFL\TE\C\AK\S
A21	*A. hydrophila*	2003	Turtle *(Mauremys mutica)*	TF3	*drfA12-orfF-aadA2*		Ile83	Ile80	AMP\S3\SXT\RD\NA\NOR\OFL\TE\DO\C\S
A27	*A. hydrophila*	2003	Turtle *(M. mutica)*	TF3	*drfA12-orfF-aadA2*		Ile83	Ile80	AMP\S3\SXT\RD\NA\TE\DO\C\S
A23	*A. hydrophila*	2003	Turtle *(M. mutica)*	TF4	*drfA12-orfF-aadA2*		Ile83	Ile80	AMP\S3\SXT\RD\NA\NOR\OFL\TE\DO\C\S
A26	*A. hydrophila*	2003	Turtle *(C. serpentine)*	TF4	*drfA12-orfF-aadA2*	*aac(6′)-Ib*	Ile83	Ile80	AMP\S3\SXT\RD\NA\NOR\OFL\TE\ DO\C\S
A03	*A. hydrophila*	1996	Fish *(Carassius auratus)*	FF1	*drfA12-orfF-aadA2*		Ile83	Ile80	AMP\S3\SXT\RD\NA\OFL\TE\DO\C\S
A19	*A.veronii*	2001	Fish *(Siniperca chuatsi)*	FF9	*drfA12-orfF-aadA2*		Ile83	Ile80	AMP\S3\SXT\NA\S
A47	*A. hydrophila*	2005	Fish *(Tenualosa reevesii)*	FF16	*drfA12-orfF-aadA2*		Ile83	Ile80	AMP\S3\SXT\RD\NA\NOR\OFL\TE\DO\C\S
A72	*A. hydrophila*	2006	Fish *(Cirrhinus molitorella)*	FF18	*drfA12-orfF-aadA2*		Ile83	Ser80	AMP\S3\SXT\NA\TE\S
A78	*A. hydrophila*	2007	Fish *(Oreochromis niloticus)*	FF22	*drfA12-orfF-aadA2*		Ile83	Ile80	AMP\S3\SXT\RD\NA\NOR\OFL\TE\DO\C\S
A01	*A. sobria*	1995	Shrimp *(Macrobrachium rosenbergii)*	SF1	*drfA12-orfF-aadA2*		Val83	Ile80	AMP\S3\SXT\RD\NA\TE\S
A41	*A. veronii*	2004	Ornamental fish *(C. auratus)*	OF2	*drfA12-orfF-aadA2, drfA17*		Val83	Ile80	AMP\S3\SXT\RD\NA\ NOR\OFL\TE\ DO\AK\S
A42	*A. veronii*	2004	Fish *(Megalobrama terminalis)*	FF11	*drfA12-orfF-aadA2, catB3, arr-3*		Ile83	Ile80	AMP\S3\SXT\RD\NA\S
A24	*A. hydrophila*	2003	Turtle *(C. serpentine)*	TF2	*drfA12-orfF-aadA2, catB3, arr-3*	*aac(6′)-Ib*	Ile83	Ile80	AMP\S3\SXT\RD\NA\CIP\NOR\OFL\TE\ DO\S
A33	*A. hydrophila*	2004	Giant salamander *(Andrias davidianus)*	AF1	*aac(6′)-II-bla*_OXA−21_*-cat3*		Ile83	Ile80	AMP\S3\SXT\NA\TE\S
A83	*A. hydrophila*	2007	Turtle *(Pelodiscus sinensis)*	TF10	*drfA12-orfF, catB3*		Ile83	Arg80	AMP\S3\SXT\RD\NA\CIP\NOR\OFL\C\S
A84	*A. hydrophila*	2007	Turtle *(Pelodiscus sinensis)*	TF10	*drfA12-orfF, catB3*	*aac(6′)-Ib-cr*	Ile83	Ile80	AMP\S3\SXT\RD\NA\CIP\NOR\OFL\C\S
A22	*A. hydrophila*	2004	Turtle *(C. serpentine)*	TF4	No insert	*bla*_TEM−1_, *aac(6′)-Ib-cr*	Ile83	Ile80	AMP\CTX\S3\SXT\RD\NA\CIP\TE\AK\S
A38	*A. hydrophila*	2004	Giant salamander *(A. davidianus)*	AF1	ND		Ile83	Ile80	AMP\S3\SXT\RD\NA\CIP\NOR\OFL\TE\ DO\C\S

*ND, not determined; FF, fish farm; OF, ornamental fish farm; TF, turtle farm; AF, amphibian farm; SF, shrimp farm; QRDR, quinolone resistance-determining regions; Ser, serine; Ile, iosleucine; Val, valine; Arg, arginine; AMP, ampicillin; CTX, cefotaxime; S3, sulfonamides; SXT, trimethoprim/sulfamethoxazole; RD, rifampin; NA, nalidixic acid; CIP, ciprofloxacin; NOR, norfloxacin; OFL, ofloxacin; TE, tetracycline; DO, doxycycline; S, streptomycin; AK, amikacin; C, chloramphenicol*.

**Figure 1:**
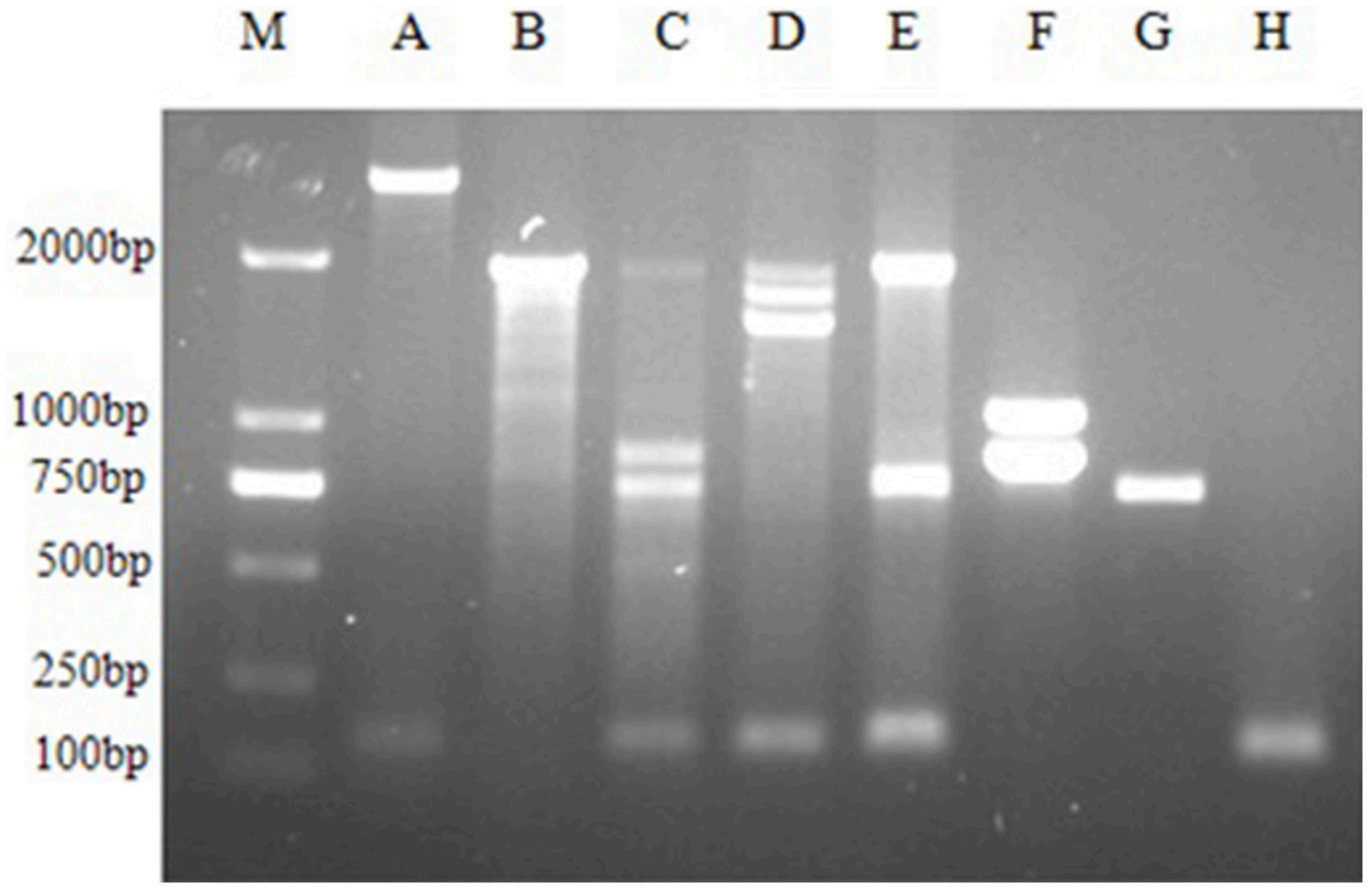
**PCR amplification products of class 1 integron variable region M: DL2000 marker; A–H: eight groups of class 1 integrons**.

The genetic content of the 22 integrons was determined through PCR amplification of the integron variable region. PCR products were obtained for 21 isolates, ranging from 750 to 2000 bp (Figure 1). One isolate was “empty” with no gene cassettes inserted between the conserved segments of the integron. The presence of six different gene cassette arrays were determined by sequencing and these were *drfA12-orfF-aadA2* (KF442255 and KF442261), *drfA12-orfF* (KF442257), *catB3* (KF442258 and KF442260), *aac(6*′*)II-bla*_*OXA*−21_*-catB3* (KM009138), *arr-3* (KF442259), and *dfrA17* (KF442256). Additionally, we identified four isolates containing two to three integrons. Thirteen isolates contained an identical restriction fragment and carried the same gene cassette arrays (*drfA12, orfF*, and *aadA2*). This was the dominant gene cassette array found in this study.

### Antimicrobial susceptibility of *Aeromonas*

The number of isolates resistant to antimicrobials was variable among the groups and 49.1% (55/112) were resistant to three or more different classes of antimicrobial agents. Resistance was most prevalent for ampicillin (85.7%), rifampin (57.1%), streptomycin (49.1%), and nalidixic acid (44.6%). Most of the isolates (≥90%) were susceptible to cefotaxime, amikacin, ciprofloxacin, norfloxacin, and doxycycline (Supplementary Table 2).

The isolates belonging to the eight identified *Aeromonas* spp. have varying levels of susceptibilities to the different antimicrobial agents. *A. hydrophila* and *A. veronii* isolates displayed greater levels of resistance as compared to other species (Supplementary Table 3). Considering the different sources, the *Aeromonas* isolates from turtles and amphibians were found to be more resistant to trimethoprim/sulfamethoxazole, quinolones, tetracyclines, and streptomycin (Supplementary Table 4).

With the exception of cefotaxime, the resistance rates of 13 antimicrobials of the integron-positive strains was significantly (*P* < 0.05) higher than the rate in integron-negative strains (Supplementary Table 2). All isolates carrying integrons were multi-drug resistant and were resistant to five or more commonly used antimicrobial agents. Resistance to more than 12 antimicrobial agents was observed in 12 isolates. The susceptibility pattern for the integron-containing strains is shown in Table 2.

### Presence of resistance genes and analysis of QRDR mutations in integron-positive strains

Among the ESBL genes, *bla*_CTX-*M*-3_ was detected in one isolate in *A. hydrophila* from fish, whereas *bla*_TEM-1_ were detected in two isolates in *A. hydrophila* from fish and turtle, and one of these isolates harbored both genes. Among the tetracycline genes, only *tetA* was present in two isolates. Five integron-harboring isolates were positive for *aac(6*′*)-Ib* but only three of them were *cr* variants associated with ciprofloxacin resistance. Moreover, two isolates were associated with both beta-lactamase and PMQR genes.

The nucleotide sequences of *gyrA* and *parC* QRDR of 22 integron-positive strains were determined *via* PCR amplification and direct sequencing. With exception of two strains with a Ser-83-Val substitution, the other strains had mutations at codon 83 resulting in a Ser to Ile change in the GyrA QRDR. In the QRDR of ParC, the substitution Ser-80-Ile was observed in 18 of 22 integron-positive strains and one isolate had a Ser-83-Arg mutation (Table 2).

## Discussion

### Characterization of integrons in *Aeromonas*

In aquaculture, disease diagnoses are often presumptive and therapeutic measures are usually administered in the absence of reliable antimicrobial resistance data. There are few antimicrobial agents that have been approved for Chinese aquaculture [Ministry of Agriculture of the People's Republic of China (MOA), 2010], so impudent and abusive use of antimicrobials is common. The emergence and dissemination of antimicrobial resistance among *Aeromonas* spp. from aquaculture has attracted much attention. As such, *Aeromonas* strains have developed resistance to various antimicrobial agents as we and others have found. Our data indicates that these resistance patterns in *Aeromonas* can be derived from integrons encoding genes conferring resistance to trimethoprim-sulfamethoxazole, streptomycin, and other agents. Multi-drug resistance mediated by integrons in *Aeromonas* may complicate future antibiotic therapies used in aquaculture as well as stimulate resistance gene transfer.

Integrons with gene cassettes found primarily in gram-negative bacteria belonging to the family Enterobacteriaceae. These are often located on conjugative plasmids or transposons which can facilitate lateral transfer between pathogens (Mazel, 2006). Recently, several studies reported the presence of integrons on similar inserted-gene cassettes in fish-pathogenic *Aeromonas* isolates with a frequency of 30–50% (Nawaz et al., 2010; Ndi and Barton, 2011; Lukkana et al., 2012). In the present study, 19.6% of *Aeromonas* isolates from farmed freshwater animals carried the class 1 integron. Taken together, the frequency of class 1 integrons in *Aeromonas* isolates from farmed freshwater animals in South China is less than those from other freshwater animals. This is most likely due to different sources of isolates and the particular background of antimicrobial usage.

The *addA2* gene responsible for resistance to streptomycin and the *dfrA12* gene encoding resistance to trimethoprim were identified in most of the integron-borne *Aeromonas* identified in this study. Both the *addA* and *dfr* genes are the most frequently found resistance genes in the variable region of integrons among different species of bacteria. A similar pattern has been reported in *Aeromonas* from aquaculture sources (Kadlec et al., 2011; Lukkana et al., 2012), environmental samples (Moura et al., 2012) as well as clinical isolates (Lee et al., 2008). The predominance of the *addA* and *dfr* genes suggests that these genes cassettes are more stable when integrated than other gene cassettes. Additionally, both the selection and dispersion of *addA* and *dfr* genes in integrons could be related to the extensive use of streptomycin and trimethoprim-sulfamethoxazole in the control of animal diseases.

We identified a new cassette combination *aac(6*′*)-II-bla*_OXA-21_*-cat3* in *A. hydrophila* isolated from a Chinese giant salamander (*Andrias davidianus*). This combination had previously only been found in *A. hydrophila* from clinical isolates in Taiwan (GenBank DQ993182.1). The Chinese giant salamander is both high medicinal value and evolutionary significance. The breeding of this animal is important in scientific research and has an economic impact as well. However, a variety of highly infectious, lethal diseases have hindered development of a breeding industry for giant salamander. The high value of these animals has motivated farmers to increase investments in antimicrobials and the same situation also appears in turtle farming and ornamental fish farming (Verner-Jeffreys et al., 2009; Kadlec et al., 2011). In this study, we also demonstrated that *Aeromonas* isolates from turtles, the Chinese giant salamander and ornamental fish were multi-drug resistant indicating that a serious problem of antimicrobial resistance may be emerging in high-value animal farming.

### Detection of other resistance genes in integron-positive isolates

We found 22 isolates containing class 1 integrons that were resistant to five or more antimicrobial agents. We then screened for genes encoding 10 other resistance determinants and four [*aac(6*′*)-Ib-cr, tetA, bla*_CTX-*M*-3_, *bla*_TEM-1_] were detected. Cephalosporins are effective human antimicrobials but are seldom used in aquaculture. Unexpectedly, we acquired one cefotaxime-resistant strain among 22 integron-positive *Aeromonas* isolates which carried *bla*_CTX-*M*-3_. Two isolates carrying *bla*_TEM-1_ were also associated with *aac(6*′*)-Ib-cr*.

The occurrence of PMQR and ESBL determinants in Enterobacteriaceae from aquatic animals has been previously demonstrated (Jiang et al., 2012). We found three *A. hydrophila* isolates from turtles (*Chelydra serpentine* and *Pelodiscus sinensis*) that harbored *aac(6*′*)-Ib-cr, bla*_TEM-1_, and/or *bla*_CTX-*M*-3_. Farmed animals with high economic values are grown over long culture cycles and farmers are seeking more effective antimicrobial agents such as ciprofloxacin, ceftriaxone, and amikacin. With the intensive use of these antimicrobial in aquaculture, the incidence of drug abuse might also post a potential risk to public health.

In conclusion, our investigation demonstrated that freshwater animals can serve as a reservoir for *Aeromonas* species containing integron-mediated multiple drug-resistant phenotypes. The association of multi-drug resistance with integrons increases the risk of co-selection and persistence of other resistance determinants under the selective pressure imposed by the use of antimicrobial agents. Therefore, prudent and responsible use of antimicrobials is a mandatory requirement to promote the healthy development of Chinese aquaculture.

## Author contributions

YD and LJ conceived the study, designed the trial, and supervised conduct of the trial. AT and LL supervised the data collection. YD and YW drafted the manuscript. RZ contributed substantially to the revision. LJ takes responsibility for the article as a whole.

### Conflict of interest statement

The authors declare that the research was conducted in the absence of any commercial or financial relationships that could be construed as a potential conflict of interest.
