# Supplementary material for: Multi-Drug Resistance Mediated by Class 1 Integrons in Aeromonas Isolated from Farmed Freshwater Animals
Source: Front Microbiol. 2016 Jun 15;7:935. doi: 10.3389/fmicb.2016.00935 (PMC4908131; doi:10.3389/fmicb.2016.00935)
Supplement: Supplementary file 1 [file Table1.PDF]

Supplementary Table1 Primers used in this study and expected sizes of PCR products

	Primer	Nucleotide sequence (5' to 3')	Size (bp)	Reference
Integrons	*intI1*-F	ATC ATC GTC GTA GAG ACG TCG G	892	Reyes A *et al.*, 2003
	intI1-R	GTC AAG GTT CTG GAC CAG TTG C		
	intI2-F	GCA AAT GAA GTG CAA CGC	467	
	intI2-R	ACA CGC TTG CTA ACG ATG		
	intI3-F	GCA GGG TGT GGA CGA ATA CG	760	
	intI3-R	ACA GAC CGA GAA GGC TTA TG		
	sulI-F	CTT CGA TGA GAG CCG GCG GC	417	Sandvang D *et al.*, 1997
	sulI-R	GCA AGG CGG AAA CCC GCG CC		
	qacE△1-F	ATC GCA ATA GTT GGC GAA GT	230	
	qacE△1-R	CAA GCT TTT GCC CAT GAA GC		
	5’CS	GGC ATC CAA GCA GCA AG	variable	Lévesque C *et al.*, 1995
	3’CS	AAG CAG ACT TGA CCT GA		
PMQR genes	*qnrA*-F	ATT TCT CAC GCC AGG ATT TG	519	Liu JH *et al.*, 2008
	qnrA-R	GAT CGG CAA AGG TCA GGT CA		
	qnrB-F	GAT CGT GAA AGC CAG AAA GG	469	
	qnrB-R	ACG ATG CCT GGT AGT TGT CC		
	qnrS-F	ACG ACA TTC GTC AAC TGC AA	417	
	qnrS-R	TAA ATT GGC ACC CTG TAG GC		
	aac(6')-Ib-F	TTG CGA TGC TCT ATG AGT GGC TA	482	
	aac(6')-Ib-R	CTC GAA TGC CTG GCG TGT TT		
	qepA-F	GCA GGT CCA GCA GCG GGT AG	306	
	qepA-R	CTT CCT GCC CGA GTA TCG TG		
tetracycline resistance genes	*tetA*-F	GTA ATT CTG AGC ACT GTC GC	956	Liu JH *et al.*, 2008
	tetA-R	CTG CCT GGA CAA CAT TGC TT		
	tetE-F	GTG ATG ATG GCA CTG GTC AT	1198	
	tetE-R	CTC TGC TGT ACA TCG CTC TT		
	tetC-F	TCT AAC AAT GCG CTC ATC GT	588	
	tetC-R	GGT TGA AGG CTC TCA AGG GC		
beta-lactamase genes	TEM-F	AAA GAT GCT GAA GAT CA	425	Liu JH *et al.*, 2008
	TEM-R	TTT GGT ATG GCT TCA TTC		
	CTX-M-F	GTG CAG TAC CAG TAA AGT TAT GG	538	
	CTX-M-R	CGC AAT ATC ATT GGT GGT GCC		
QRDRs	*gyrA*-F	CCA TGA GCG TGA TCG TAG GA	665	Giraud E *et al.*, 2004
	gyrA-R	CTT TGG CAC GCA CAT AGA CG		
	parC-F	GTT CAG CGC CGC ATC ATC TAC	245	
	parC-R	TTC GGT GTA ACG CAT TGC CGC		
