# Supplementary material for: Multi-Drug Resistance Mediated by Class 1 Integrons in Aeromonas Isolated from Farmed Freshwater Animals
Source: Front Microbiol. 2016 Jun 15;7:935. doi: 10.3389/fmicb.2016.00935 (PMC4908131; doi:10.3389/fmicb.2016.00935)
Supplement: Supplementary file 2 [file Table2.PDF]

Supplementary Table2 Antimicrobial susceptibilities of 112 *Aeromonas* isolates and comparison of antimicrobial resistance between integron-positive and integron-negative isolates

Antimicrobials	Breakpoints[a] (mm)		Percentage (no.) of strains resistant			χ2	*P*-value[b]
	S	R	all isolates （n=112）	integron positive （n=22）	integron negative（n=90）		
Ampicillin	≧17	≦13	85.7 (96)	100 (22)	82.2 (74)	3.227	0.023
Cefotaxime	≧23	≦14	2.7 (3)	9.1 (2)	1.1 (1)	1.800	0.098
Amikacin	≧17	≦14	3.6 (4)	13.6 (3)	1.1 (1)	4.827	0.023
Streptomycin	≧15	≦11	49.1 (55)	100 (22)	36.7 (33)	25.896	0.000
Rifampin	≧20	≦16	57.1 (64)	86.4 (19)	50.0 (45)	8.118	0.001
Nalidxic acid	≧20	≦14	44.6 (50)	100 (22)	31.1 (28)	31.218	0.000
Ciprofloxacin	≧21	≦15	5.4 (6)	27.3 (6)	0	20.835	0.000
Norfloxacin	≧17	≦12	9.8 (11)	50.0 (11)	0	44.415	0.000
Ofloxacin	≧25	≦21	13.4 (15)	59.1 (13)	2.2 (2)	44.510	0.000
Sulfonamides	≧17	≦12	31.2 (35)	100 (22)	13.3 (12)	58.775	0.000
Trimethoprim /sulfamehtoxazole	≧16	≦10	21.4 (24)	100 (22)	2.2 (2)	105.754	0.000
Tetracycline	≧19	≦14	28.6 (32)	81.8 (18)	15.6 (14)	34.857	0.000
Doxycycline	≧14	≦10	12.5 (14)	45.5 (10)	4.4 (4)	23.564	0.000
Chloramphenicol	≧18	≦12	13.4 (15)	63.6 (14)	1.1 (1)	51.316	0.000

[a] CLSI (2006a,b).

[b] Differences in resistance between the two groups were established using the χ[2] test and were considered significant at a *P* value of ＜0.05.
