# Supplementary material for: Multi-Drug Resistance Mediated by Class 1 Integrons in Aeromonas Isolated from Farmed Freshwater Animals
Source: Front Microbiol. 2016 Jun 15;7:935. doi: 10.3389/fmicb.2016.00935 (PMC4908131; doi:10.3389/fmicb.2016.00935)
Supplement: Supplementary file 3 [file Table3.pdf]

Supplementary Table 3 Comparison of antimicrobial resistance between different species of *Aeromonas* spp.

Antimicrobial agents	Percentage (no.) of strains resistant								
	A. hydrophila (n=62)	*A. veronii* (n=23)	*A. sobria* (n=6)	*A. caivae* (n=7)	*A. dhakensis* (n=4)	*A. trota* (n=4)	*A. jandaei* (n=3)	*A. media* (n=1)	unidentified (n=2)
Ampicillin	95.2 (59)	100.0 (23)	100.0 (6)	0	100.0 (4)	0	100.0(3)	100.0 (1)	0
Cefotaxime	3.2 (2)	4.3 (1)	0	0	0	0	0	0	0
Sulfonamides	35.5 (22)	52.2 (12)	16.7 (1)	0	0	0	0	0	0
Trimethoprim/ sulfamethoxazole	29.0 (18)	21.7 (5)	16.7 (1)	0	0	0	0	0	0
Rifampin	53.2 (33)	60.9 (14)	66.7 (4)	71.4 (5)	75.0 (3)	100.0 (4)	0	100.0 (1)	0
Nalidixic acid	41.9 (26)	78.3 (18)	33.3 (2)	28.6 (2)	25.0 (1)	25.0 (1)	0	0	0
Ciprofloxacin	8.1 (5)	4.3 (1)	0	0	0	0	0	0	0
Norfloxacin	16.1 (10)	4.3 (1)	0	0		0	0	0	0
Ofloxacin	19.4 (12)	13.0 (3)	0	0	0	0	0	0	0
Tetracycline	32.3 (20)	34.8 (8)	33.3 (2)	14.3 (1)	0	25.0 (1)	0	0	0
Doxycycline	19.4 (12)	13.0 (3)	0	0	0	0	0	0	0
Streptomycin	37.1 (23)	73.9 (17)	83.3 (5)	42.9 (3)	50.0 (2)	50.0 (2)	66.7(2)	0	50.0 (1)
Amikacin	3.2 (2)	8.7 (2)	0	0	0	0	0	0	0
Chloramphenicol	22.6 (14)	4.3 (1)	0	0	0	0	0	0	0
