# Supplementary material for: Multi-Drug Resistance Mediated by Class 1 Integrons in Aeromonas Isolated from Farmed Freshwater Animals
Source: Front Microbiol. 2016 Jun 15;7:935. doi: 10.3389/fmicb.2016.00935 (PMC4908131; doi:10.3389/fmicb.2016.00935)
Supplement: Supplementary file 4 [file Table4.PDF]

Supplementary Table 4 Comparison of antimicrobial resistance between different sources

Antimicrobial agents	Percentage (no.) of strains resistant				
	amphibians (n=5)	turtles (n=21)	Ornamental fish (n=7)	fish (n=67)	Shrimps (n=12)
Ampicillin	80.0 (4)	100 (21)	100.0 (7)	89.6 (60)	33.3 (4)
Cefotaxime	20.0 (1)	9.5 (2)	0	0	0
Sulfonamides	60.0 (3)	57.1 (12)	42.9 (3)	23.9 (16)	8.3 (1)
Trimethoprim/sulfamethoxazole	60.0 (3)	47.6 (10)	28.6 (2)	11.9 (8)	8.3 (1)
Rifampin	80.0 (4)	76.2 (16)	57.1 (4)	44.8 (30)	83.3 (10)
Nalidixic acid	60.0 (3)	71.4 (15)	57.1 (4)	34.3 (23)	41.7 (5)
Ciprofloxacin	20.0 (1)	19.0 (4)	14.3 (1)	0	0
Norfloxacin	20.0 (1)	33.3 (7)	14.3 (1)	3.0 (2)	0
Ofloxacin	20.0 (1)	38.1 (8)	28.6 (2)	6.0 (4)	0
Tetracycline	60.0 (3)	47.6 (10)	71.4 (5)	14.9 (10)	33.3 (4)
Doxycycline	40.0 (2)	33.3 (7)	28.6 (2)	4.5 (3)	0
Streptomycin	60.0 (3)	57.1 (12)	28.6 (2)	52.2 (35)	25.0 (3)
Amikacin	20.0 (1)	9.5 (2)	14.3 (1)	0	0
Chloramphenicol	40.0 (2)	33.3 (7)	14.3 (1)	7.5 (5)	0
